# The DoDo experience: an alternative antiretroviral 2-drug regimen of doravirine and dolutegravir

**DOI:** 10.1007/s15010-023-02075-y

**Published:** 2023-08-01

**Authors:** Stefanie Sammet, Veronique Touzeau-Römer, Eva Wolf, Pia Schenk-Westkamp, Birgit Romano, Elke Gersbacher, Ulrich Kastenbauer, Christoph Boesecke, Jürgen Rockstroh, Stefan Scholten, Stephan Schneeweiss, Julia Roider, Ulrich Seybold

**Affiliations:** 1 Klinik für Dermatologie und Venerologie, Universitätsklinikum Essen, Hufelandstrasse 55, 45122 Essen, Germany; 2 Universitätsklinik für Dermatologie, Medizinische Universität Wien, Währinger Gürtel 18-20, 1090 Vienna, Austria; 3 MUC Research GmbH, Waltherstr. 32, 80337 Munich, Germany; 4 Infektiologie Schwabing, Ainmillerstr. 26, 80801 Munich, Germany; 5 Medizinische Klinik und Poliklinik I, Universitätsklinikum Bonn, Campus-Venusberg, Gebäude 26, 53127 Bonn, Germany; 6 Praxis Hohenstaufenring, Richard-Wagner-Str. 9-11, 50674 Cologne, Germany; 7 Sektion Klinische Infektiologie, Medizinische Klinik und Poliklinik IV, LMU Klinikum, Ludwig-Maximilians-Universität München, Pettenkoferstr. 8a, 80336 Munich, Germany; 8 German Center for Infection Research, Partner Site Munich, Munich, Germany

**Keywords:** Antiretroviral therapy, 2-drug regimen, Dolutegravir, Doravirine, HIV

## Abstract

**Background:**

Currently available antiretroviral 2-drug regimen (2DR) fixed dose combinations may not be suitable for specific situations including the presence of resistance associated mutations (RAM) or drug − drug interactions (DDI). The data on the use of the non-nucleoside reverse transcriptase inhibitor doravirine (DOR) and the integrase inhibitor dolutegravir (DTG) as an alternative 2DR remain scarce.

**Methods:**

People living with HIV with DOR + DTG as a 2DR are being followed in a prospective observational study.

**Results:**

This analysis describes 85 participants with a median age of 57 years. Median CD4-nadir was 173/µl and a majority (66%) had a history of HIV-associated or AIDS-defining conditions. Antiretroviral history was mostly extensive, and documentation of RAM was frequent. The main reasons for choosing DOR + DTG were DDI (29%), tolerability (25%), and cardiovascular risk reduction (21%). Plasma viral load at switch was < 50 copies/ml in all but 3 instances, median CD4 count was 600/µl. DOR + DTG was later changed to another regimen in 10 participants after a median of 265 days, the other 75 participants have remained on DOR + DTG for a median of 947 days.

**Conclusion:**

DOR + DTG as a 2DR proved to be a durable treatment option even in extensively pretreated individuals.

## Introduction

People living with HIV/AIDS (PLWH) with access to antiretroviral therapy (ART) suppressing plasma viral load (VL) have an almost normal life expectancy [1]. Therefore, in current treatment guidelines [2–4] side effect profiles play a major role for consideration of specific drugs. In addition to improved tolerability of individual substances, the increasing use of two-drug regimens (2DR) instead of 3-drug combinations might reduce long-term toxicity [5].

A fixed-dose combination (FDC) 2DR of the nucleoside reverse transcriptase-inhibitor (NRTI) lamivudine (3TC) and the integrase-inhibitor (INI) dolutegravir (DTG) is considered a first-line recommendation for PLWH starting ART [2–4]. However, it is not approved in case of resistance to either 3TC or DTG including detection of the reverse transcriptase (RT) resistance-associated mutations (RAM) M184V or M184I, present in up to a third of ART experienced PLWH in Germany [6]. An association of M184V with virological failure of 3TC/DTG in individuals with shorter preceding time of virological suppression has been reported [7].

Another 2DR combining the non-NRTI (NNRTI) rilpivirine (RPV) again with DTG can be an attractive option even for PLWH with extensive treatment history. Although RAM limiting the use of RPV/DTG are far less frequent, drug − drug interactions (DDI), e.g. with gastric acid reducing medication including proton pump inhibitors (PPI) [8] frequently preclude the inclusion of RPV as part of an ART regimen. In addition, its additive effect on QTc prolongation may be prohibitive.

The next-generation NNRTI doravirine (DOR) was introduced in 2018 and is available both as single drug for combination with other antiretrovirals and as 3-drug FDC with tenofovir-DF (TDF) and 3TC. Solid evidence for its safe and effective use in both contexts is available from several randomized trials [9–11], and doravirine now also is among the recommended drugs for first-line ART in Europe [2, 3]. Doravirine has little cross resistance to other NNRTI in addition to a relatively high resistance barrier [12] and thus can be an option also for PLWH with extensive ART history. Its potential for DDI is low including compatibility with gastric acid reducing agents and it is suitable for taking both with and without food [13]. In addition, concomitant administration of DOR and DTG has been shown to cause no clinically significant alteration in the pharmacokinetic and safety profiles of either drug [14].

However, despite being a theoretically attractive and both FDA- and EMA-approved combination, DOR + DTG has not been specifically studied in any randomized trial. Even observational data remain scarce [15, 16]. The objective of this study was to establish a first “real-world” evidence base for the use of DOR + DTG as a 2DR.

## Methods

All adult PLWH whose ART was switched to a 2DR consisting exclusively of DOR + DTG at 4 university outpatient clinics and 3 private practices in Germany and Austria were asked to participate in this ongoing observational study. Written informed consent was obtained from all participants, the respective ethics committees approved the study (project nr. 21–0044 at LMU Klinikum Munich). Demographic and medical data were abstracted from charts and electronic records with a planned annual follow-up of a maximum of 5 years after the switch to DOR + DTG. This brief report provides the descriptive analysis of patient characteristics at the time of switch to DOR + DTG as well as a preliminary assessment of durability of this 2DR.

## Results

Through April 2023, 85 PLWH with a previous ART-switch to DOR + DTG have been included in the study (Table 1). The switch occurred between February 2019 and March 2023, resulting in a total of 2207 person-months on DOR + DTG. The median age at the time of switch was 57 years (range 19–81), female/male ratio was 18/67 (21%/79%). HIV-associated or AIDS-defining conditions had previously been diagnosed in 56 participants (66%), the median CD4-nadir was 173/µl (range 0–922). The vast majority had an extensive ART history, with RPV/DTG as directly preceding regimen in 22 individuals (26%).

**Table 1:** Characteristics of 85 people living with HIV/AIDS at the time of switch to a 2-drug-regimen consisting of DOR + DTG (doravirine + dolutegravir, “DoDo”)

Characteristic	Total *N* = 85	*n*	(%)
*Gender*			
	Female	18	21%
	Male	67	79%
*Age [years]*			
	(median (range))	57	(19–81)
*Medical history*			
	HIV-associated condition	24	28%
	AIDS	32	38%
	CD4-T-cell-count nadir < 200 cells/µl	55	65%
CD4-T-cell count nadir [cells/µl]	(median (range))	173 (0–922)	
Years on ART	(median (range))	21 (0–34)	
Number of previous ART regimens	(median (range))	6 (1–22)	
*ART experience*			
	NRTI	85	100%
	NNRTI	75	88%
	INSTI	73	86%
	PI	70	82%
	Maraviroc	6	7%
	Enfuvirtide	2	2%
*Switch from*			
	Nucleos/tide sparing regimen	52	61%
	INSTI-containing regimen	70	82%
	Including dolutegravir	53	62%
	Rilpivirine/dolutegravir	22	26%
	Dolutegravir + darunavir	10	12%
	Lamivudine/dolutegravir	4	5%
	Including raltegravir	9	11%
	Including bictegravir	5	6%
	Including elvitegravir/cobicistat	2	2%
	Including cabotegravir	1	1%
	NNRTI-containing regimen	40	47%
	Including rilpivirine	24	28%
	Including etravirine	7	8%
	Including nevirapine	5	6%
	Including efavirenz	2	2%
	Including doravirine	2	2%
	PI-containing regimen	31	36%
	Including darunavir	27	32%
	Including atazanavir	3	4%
	Including lopinavir/ritonavir	1	1%
*Main reason for switch (multiple selection)*			
	Drug − drug interactions	25	29%
	Gastric-acid reducing agents	18	21%
	Side-effects of previous regimen	21	25%
	Cardiovascular risk reduction	18	21%
	Convenience/pill burden	10	12%
	Renal function	8	9%

*ART* antiretroviral therapy, *NRTI* nucleoside/nucleotide reverse transcriptase inhibitors, *NNRTI* non-nucleoside reverse transcriptase inhibitors, *PI* protease inhibitors, *INSTI* integrase strand transferase inhibitors

For 66 participants (77.6%) (cumulative) genotypic resistance testing data were available, in 9 instances (10.6%) resistance testing had never been performed, and for 10 others (11.8%) information on prior resistance testing was unavailable. Protease-RAM were documented in 32 cases (38%), NRTI-RAM in 43 (51%). For 30 participants (35%) RT-RAM were considered to affect NNRTI. For 21 participants (25%) the German GRADE 01/2023 algorithm [17] identified so-called flagged mutations but still considered the virus as susceptible to DOR. In one participant the V106A mutation was later identified from an historic genotype, indicating (high-level) resistance to DOR. The combination of the NNRTI-RAM Y181V and F227L in another participant was scored as resistance by the HIVDB 9.4 algorithm, the combination of K103N and Y181C in a third patient was scored as resistance by ANRS 33_10/2022. Both combinations were scored as flagged mutations/susceptible by GRADE [17].

In 2 participants (2%) integrase RAM scored by prediction algorithms had been documented. The presence of N155H resulted in the use of DTG 50 mg twice daily for one patient, in the other the T74I and T97A mutations were not considered to significantly impair the effect of DTG [17]. An overview of resistance data including interpretations of genotypes by the GRADE-, ANRS-, HIVDB-, and Rega algorithms for DOR and DTG are shown in Table 2.

**Table 2:** Information on cumulative genotypic resistance testing among 85 people living with HIV/AIDS at the time of switch to a 2-drug-regimen consisting of DOR + DTG (doravirine + dolutegravir, “DoDo”)

Total *N* = 85	*n*	(%)
*Resistance testing information*		
Results available	66	77.6%
Testing never performed	9	10.6%
No information available	10	11.8%
*Reverse transcriptase*		
Any mutation documented	57	67%
RAM affecting currently approved NRTI	43	51%
RAM affecting currently approved NNRTI	30	35%
DOR-RAM: algrothm-specific interpretation		
GRADE 01/2023^a^		
Flagged mutation(s) (S)	21	25%
Resistance (R)	1	1%
ANRS 33_10/2022^a^		
Possible resistance (I)	4	5%
Resistance (R)	2	2%
HIVDB 9.4^a^		
Low level/intermediate resistance (score 15–40) (I)	9	11%
High level resistance (score ≥ 60) (R)	2	2%
*Integrase*		
Any mutation documented	4	5%
RAM affecting DTG	2	2%
N155H (algorithm-specific interpretation:)	1	1%
GRADE 01/2023^a ^	DTG qd: Intermediate (I), DTG bid: Flagged mutation (S)	
ANRS 33_10/2022^a^	DTG qd: Resistance (R), DTG bid: Susceptible (S)	
HIVDB 9.4^a^	Potential Low-Level Resistance (Score: 10) (S)	
Rega 10.0.0^a^	Susceptible GSS 1 (Score: 0.5) (S)	
L74I, T97A﻿ (algorithm-specific interpretation:)	1	1%
GRADE 01/2023^a^	Flagged mutations (S)	
ANRS 33_10/2022^a^	Susceptible (S)	
HIVDB 9.4^a^	Susceptible (S)	
Rega 10.0.0^a^	Susceptible GSS 1 (Score: 0.25) (S)	
*Protease*		
Any mutation reported	48	56%
RAM affecting currently approved PI	43	51%
*Tropism testing*		
Test results available	*N* = 16	(%)^b^
CCR5	9	56%
Dual/mixed	1	6%
CXCR4	6	38%

*RAM* resistance associated mutation, *NRTI* nucleoside/nucleotide reverse transcriptase inhibitors, *NNRTI* non-nucleoside reverse transcriptase inhibitors, *DOR* doravirine, *GRADE* Genotypic Resistance-Algorithm Deutschland [17]. (S), susceptible, (*R*) resistance, *ANRS* Agence Nationale de Recherche sur le Sida resistance algorithm, (*I*) intermediate, *HIVDB* Stanford HIV drug resistance database, *DTG* dolutegravir, *qd* once daily, *bid* twice daily, *Rega* Rega institute HIV-1 subtyping tool, *PI* protease inhibitors, *CCR5* C–C-motif chemokine receptor type 5, *CXCR4* C-X-C-motif chemokine receptor type 4

^a^All genotypic resistance interpretation algorithms were accessed via the GRADE website[17]

^b^Based on the observed data

The main reasons for the switch to DOR + DTG were tolerability of the preceding regimen (*n* = 21, 25%), DDI (*n* = 25, 29%) and specifically gastric acid inhibiting agents (*n* = 18, 21%), as well as reduction of cardiovascular risk (*n* = 18, 21%); 44 participants (52%) received gastric acid reducing agents at the time of switch. Plasma viral load (VL) approximately one year before the switch was < 50 copies/ml in all but one participants and at the time of switch was < 50 copies/ml in all but 3 participants, median CD4 count at the time of switch was 600/µl (range 110–1873). In all 3 instances where the switch to DOR + DTG was made in the presence of detectable viremia of 80, 750, and 160,000 copies/ml, respectively, VL was resuppressed at the next available follow-up while on DOR + DTG.

During the follow-up, ART was again changed from DOR + DTG to another regimen in 10 participants (12%) after a median of 265 days (range 20–1390): 5 patients continued to experience potentially ART-associated side effects despite the switch to DOR + DTG, one wished to return to a single tablet regimen of RPV/DTG after stopping pantoprazole, and one wished to switch to long acting injectable ART. For one participant the identification of the DOR-RAM V106A in an historic genotypic resistance test led to the recommendation to switch off DOR + DTG despite a continuously undetectable VL. In 2 participants persisting low-level viremia of < 100 copies/ml led to the add-on of lamivudine. The other 75 participants (88%) have remained on DOR + DTG for a median of 947 days (range 43–1543) with no occurrence of virologic failure. While on DOR + DTG, 4 male participants died from non-AIDS comorbidities at age 62, 63, 66, and 82, respectively.

## Discussion

PLWH whose ART was switched to a 2DR of DOR + DTG had a median age of 57 years and thus were significantly older than the median of about 50 years among PLWH in Germany [18]. The majority had been living with HIV for more than 24 years and been on ART for more than 21 years. With a median number of 6 and up to 22 previous ART regimens almost all of them are considered extensively pretreated. The majority had also experienced HIV-associated or even AIDS-defining illnesses.

Despite this, the switch to DOR + DTG mostly occurred in an immunologically stable situation with only 9 individuals (11%) having a CD4-count of < 350/µl at the time of switch, which never occurred due to virological failure of the previous regimen. The main reasons for the switch were rather the attempt to optimize ART with respect to current treatment-associated adverse effects and DDI, specifically compatibility with a PPI, and the intent to reduce the risk for potential future complications of ART. To achieve this, physicians resorted to the off-label prescription of DOR in 31 cases (36%) due to the presence of NNRTI-RAM.

Although the majority of participants still continue on DOR + DTG, 10 individuals (12%) have switched to other regimens. So far, only 2 instances of low level viremia (already present throughout the year before the switch to DOR + DTG) have been observed among this group of individuals living with HIV, confirming the high barrier to resistance expected for this combination. In all instances tolerability issues resulting in switching off DOR + DTG represented persistence of symptoms already present during previous ART. While for 10 participants the convenience of 2 pills without food requirements was the main reason to choose DOR + DTG, 2 others preferred more convenient options (single tablet regimen and injectables) as soon as these became available to them.

The characteristics of PLWH on DOR + DTG observed in this study indicating older ager and extensive previous ART experience, the main reasons for switching, as well as the so far favorable course of treatment are in accordance with anecdotal reports and also two smaller observational studies of 18 PLWH from Italy [16] and 12 PLWH from Washington, DC [15] who switched to a 2DR of DOR + DTG, all at ≥ 50 years of age.

The limitations of our study include its observational nature and obvious potential for selection bias. We believe; however, that the inclusion of PLWH from university hospital clinics as well as private practices allows for the documentation of representative real-life treatment situations in Austria and Germany. The predominance of patients from university hospital clinics may also reflect the complex treatment situation of patients on DOR + DTG. The still limited number of participants and the absence of a control group both preclude a comparative analysis, e.g., with other ART regimens. Given the extensive and diverse history of ART and HIV-associated complications among most participants, any such analysis will require a larger number of participants to allow adjustment for confounding variables. Therefore, we focused on the description of patient characteristics and their respective reasons for switching to DOR + DTG.

To our knowledge, this is the largest published report on the use of DOR + DTG as a 2DR so far. Both inclusion of additional participants from other institutions and further analysis of follow-up data are planned.

## Conclusion

A 2DR of DOR + DTG (“DoDo”) can be a valuable option for optimizing ART also in PLWH with extensive treatment history. Further study of this combination with a high barrier to resistance and low potential for DDI is clearly warranted.

## Data Availability

Owing to the data protection requirements for this ongoing study data are not publicly available.
